# (Radio)Biological Optimization of External-Beam Radiotherapy

**DOI:** 10.1155/2012/329214

**Published:** 2012-11-06

**Authors:** Alan E. Nahum, Julien Uzan

**Affiliations:** Physics Department, Clatterbridge Cancer Centre, Bebington CH63 4JY, UK

## Abstract

“Biological optimization” (BIOP) means planning treatments using (radio)biological criteria and models, that is, tumour control probability and normal-tissue complication probability. Four different levels of BIOP are identified: Level I is “isotoxic” individualization of prescription dose *D*_presc_ at fixed fraction number. *D*_presc_ is varied to keep the NTCP of the organ at risk constant. Significant improvements in local control are expected for non-small-cell lung tumours. Level II involves the determination of an individualized isotoxic combination of *D*_presc_ and fractionation scheme. This approach is appropriate for “parallel” OARs (lung, parotids). Examples are given using our BioSuite software. Hypofractionated SABR for early-stage NSCLC is effectively Level-II BIOP. Level-III BIOP uses radiobiological functions as part of the inverse planning of IMRT, for example, *maximizing TCP whilst not exceeding a given NTCP*. This results in non-uniform target doses. The NTCP model parameters (reflecting tissue “architecture”) drive the optimizer to emphasize different regions of the DVH, for example, penalising high doses for quasi-serial OARs such as rectum. Level-IV BIOP adds functional imaging information, for example, hypoxia or clonogen location, to Level III; examples are given of our prostate “dose painting” protocol, *BioProp*. The limitations of and uncertainties inherent in the radiobiological models are emphasized.

## 1. Introduction

### 1.1. Advances in Radiotherapy Technology and Practice from the 1960s to the Present

The practice of external-beam radiation therapy is heavily influenced by the technology available in any one era. In the 1960s linear accelerators began to replace Cobalt machines [1]. Thus higher (bremsstrahlung) photon energies with improved tissue penetration became available. The next major advance was computerized treatment planning systems (TPSs) and shortly thereafter computer tomography (CT) provided 3-D images of the patient anatomy which were imported into the TPS [2]. Through advances in dose computation “algorithms” for megavoltage photon beams, the first planning systems worthy of the adjective “3-D” were developed and began to be available commercially [3]. These early 3-D TPSs made vastly improved targeting of the tumour volume possible but also revealed just how much normal tissue was irradiated using the crude collimation techniques of the time. Beam's eye view (BEV) computer graphics then led to much improved beam shaping, firstly through custom casting of shielding blocks (which became standard practice in N. America in the 1980s) and subsequently via the computer-controlled multileaf collimator [4]. Already at this stage the improved sparing of normal tissues encouraged efforts at “dose escalation” [5]. However, the really major advance was intensity modulation (IMRT) with its associated “inverse planning” which took normal-tissue sparing or “sculpting” to a whole new level [6–8]. One could now ask the TPS to create a treatment plan which significantly reduced the volume of high dose to even “concave” organs at risk (OAR) situated tightly adjacent to the target volume (in practice the PTV), for example, rectum to the prostate gland. More generally, a vastly increased number of degrees of freedom in the inverse planning process were now available. And the onward march of technology shows no signs of abating. Tomotherapy Hi-Art (Accuray) and more recently RapidArc (VARIAN) and VMAT (Elekta) have provided practical solutions for converting multifield IMRT into continuously rotating intensity modulation, originally known as IMAT [9], making IMRT easier and faster to deliver. In parallel with these advances in beam delivery and treatment planning, on-couch patient imaging was being steadily developed, from the early EPIDS [10] through to kV cone-beam CT [11] which is driving “image guidance” or IGRT. In addition to all this increasing sophistication of megavoltage-*photon* techniques, gantry-based, high-energy, scanned-beam proton therapy is now becoming available in the more affluent parts of the world, principally the USA, Japan, and Europe, thereby achieving even higher degrees of “conformality” and hence greater normal tissue sparing than is possible with any photon-beam technique [12–15].

### 1.2. How Tumour Dose Is Prescribed

The advances in technology described above have been translated into increases in dose to the tumour, but this has largely been by a “one-size-fits-all” strategy, that is, *escalate the (prescription) dose D*_presc_ *to the same value for every patient *(*e.g., due to the adoption of IMRT, protons, etc.) or change the number of fractions in the same way for each patient (e.g., prostate or breast hypofractionation due to low tumour* *α*/*β*).

Despite the vastly increased normal-tissue-sparing and tumour-targeting ability at our disposal, the way in which the dose to the tumour is “prescribed” has hardly changed at all. With very few exceptions, strict protocols are followed. These specify for a given tumour type the precise radiation dose that shall be delivered, within tightly defined uniformity limits (e.g., the PTV shall be encompassed by the 95% isodose), in a fixed number of fractions (delivered daily between Monday and Friday). This rigid predetermination of the very quantities, the total and fractional doses to the tumour, *D*_presc_ and *d*_presc_, that determine the probability of local control, removes the single most important weapon for improving treatment outcome—increasing the tumour dose and/or fraction size.

The above dose-escalation strategy has resulted in some modest gains in local control rates, particularly for intermediate- and late-stage prostate tumours [16] for no increase or even decreases in (principally rectal) complication rates. But as will be demonstrated in what follows, a rigid predefined dose prescription will lead to underdosage in some patients and to adverse effects from overdosage in others [17]. With reference to the different levels of biological optimization described in what follows, we label the above approach “level-zero optimization.”

## 2. Materials and Methods

### 2.1. Radiobiological Models for TCP and NTCP

Models for estimating the probability of tumour (local) control (TCP) and of normal-tissue complication (NTCP) were first proposed in the second half of the 1980s and the first half of the 1990s (see [18] for a useful summary of the various models and associated references). In the intervening period of 20 years or so the active use of these models has largely been confined to *evaluating* treatment plans, despite a very considerable literature on the analysis of clinical outcome data for determining “best fit” parameter values, recently summarized by the QUANTEC project on normal tissue complications [19]. By definition these best-fit parameters make the models reproduce the clinical data points they were fitted to, but the associated confidence intervals are usually fairly wide. The source of this sometimes large uncertainty may reside in the functional form of the model but also in the nature of the data and the way they are reported. However, it should be borne in mind that uncertainty is intrinsic to radiotherapy treatments; for example, meeting normal tissue dose criteria is not a guarantee against the occurrence of a complication. To some extent, biological models are also subject to this uncertainty. All the radiobiological evaluations given in this paper should be understood as estimates based on the best-fit parameters available today. 

It is emphasized that the current TCP, NTCP models are hybrid in nature; they apply to the *individual* patient's dose distribution, expressed in terms of dose-volume histograms (DVHs), but to the *population*-averaged patient biology. Because in general we do not know the radiosensitivity of the tumour clonogens of the patient in question [20], the “Marsden” TCP model [21, 22] uses a mean *α* and an assumed standard deviation *σ*_*α*_ over the population; how these parameters are obtained for a given tumour type is briefly described in the next section. The currently used NTCP models also involve population-averaged biology though this is generally implicit rather than explicit in their mathematical form.

Certain radiobiological models are wholly or partly mechanistic (e.g., Marsden TCP, Relative Seriality [18]) while others are purely phenomenological (Lyman [23] and Kutcher et al. [24]). In all cases, some assumptions are explicitly or implicitly made so as to render the problem of predicting the outcome of radiotherapy mathematically manageable. For example, the LKB model considers all volume elements of a particular organ to have the same importance for the function of this organ. Furthermore the 3-D dose distribution in the organ/tissue is represented by a dose-volume histogram, which is inherently 2-D and does not include any spatial information. The Marsden TCP model assumes firstly that a tumour is only “controlled” (i.e., eliminated) when every single clonogen has been “killed” (i.e., rendered incapable of further division), and further, at least as applied in the examples given here, that all the clonogens have the same radiosensitivity and that this remains constant from fraction to fraction. It is important to keep in mind these assumptions.

**Figure 1:**
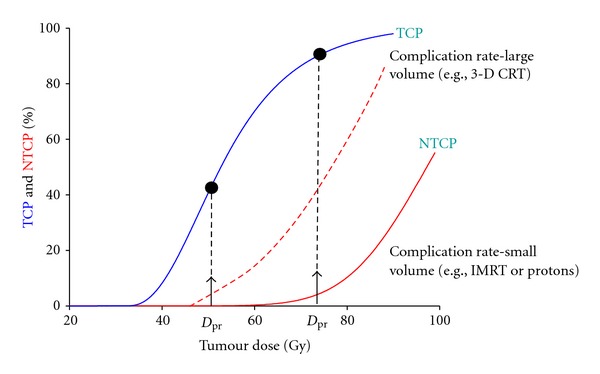
Illustration of the potential of TCP/NTCP-based optimization; the two arrows on the “Tumour Dose” axis indicate two different “isotoxic” prescription doses, *D*_pr_, associated with the full and dashed NTCP curves which correspond to “large volume” and “small volume” dose coverage of the OAR. The improvement in TCP, from *≈*45% to *≈*90%, that would result from such a change in dose to the tumour, is also shown.

In the present paper our focus is firmly on using NTCP and TCP models to change or *optimize *the way that (external-beam) treatment planning is done. The *power* of this approach is illustrated by Figure 1. However, in cases where metrics such as EUD for tumours [25, 26], gEUD for normal tissues [27], and mean lung dose (MLD) [28] are closely correlated with either TCP or NTCP then optimization based on these surrogate quantities can also be classed as *radiobiological optimization.*

### 2.2. The Different “Levels” of Radiobiological Optimization

Radiobiological models can be used to optimize treatment plans in a variety of ways. We have found it convenient to define five different “levels,” in approximate order of increasing sophistication:

**Level I**

Individualisation of *D*_presc_ on an *isotoxic *(i.e., iso-NTCP) basis.

**Level II**

Individualisation of not only *D*_presc_ but also the *number of fractions *on an *isotoxic* basis.

**Level III**

The use of radiobiological functions (EUD and/or NTCP and TCP) in the *inverse planning *algorithm.

**Level IV**

Additionally, patient-specific information from functional imaging is added to radiobiological inverse planning dose-painting.

**Level V**

Individual patient biology, for example from genomics, is added to any of the above.

Levels I to IV will be discussed in turn and illustrated by examples generated using software created at our centre.

## 3. Results and Discussion

### 3.1. Isotoxic Prescription Dose Customization: Level I

Shortly after the appearance of the first NTCP models [23, 24, 29] it was suggested that these models could be used to choose an optimum dose to the tumour [30, 31]; this was followed by more detailed studies such as [32]. However, it is only relatively recently that “isotoxic” clinical protocols have been put into practice [33]. 

**Figure 2:**
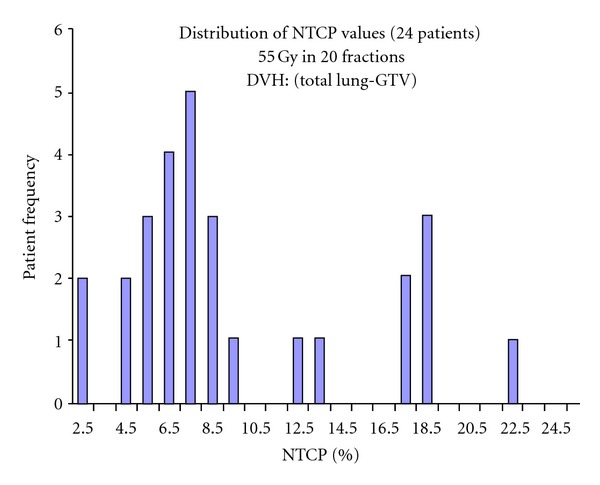
The distribution of NTCP values (grade 2 pneumonitis) estimated for a series of Clatterbridge NSCLC patients all with a *D*_presc_ of 55 Gy in 20 fractions; LKB model used with parameters *α*/*β* = 3; *TD*_50_ = 24.5 Gy*; m* = 0.37, *n* = 1 [35]. The extremely wide variation in NTCP is simply a reflection of the wide variation in tumour sizes, tumour position, and hence volume of lung in the radiation fields. Note that the average NTCP was 9.5% [36] (adapted from [38]).

**Figure 3:**
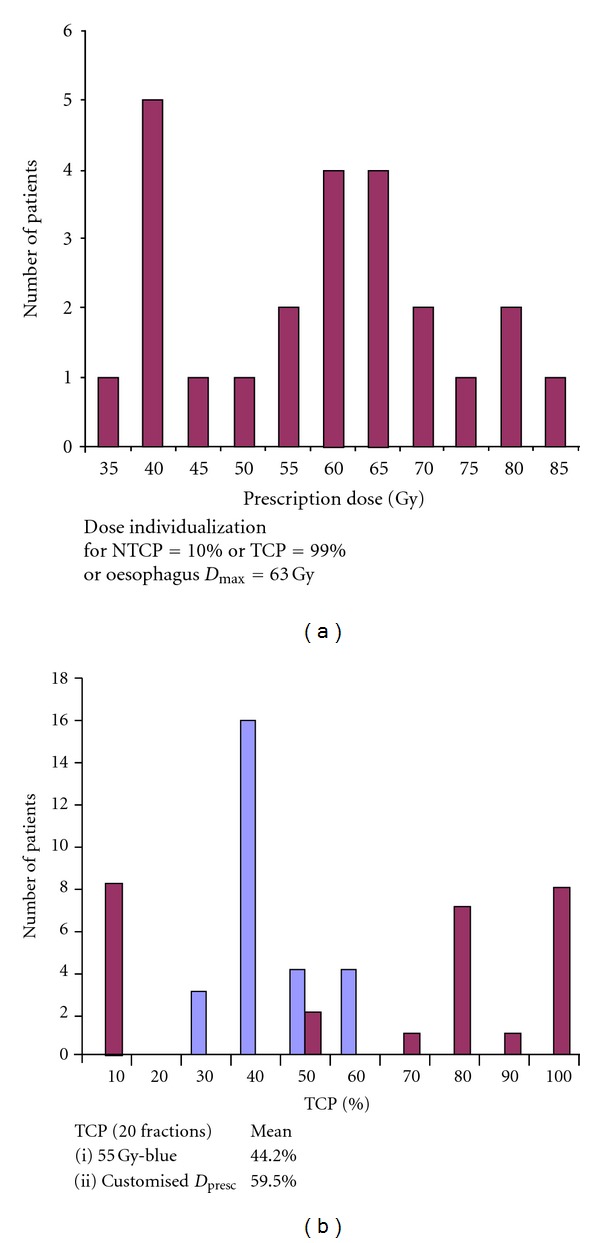
(a) The spectrum of *D*_presc_ resulting from “isotoxic” NTCP = 10% (grade 2 radiation pneumonitis) or TCP = 99% or *D*_max⁡_(oesophagus) = 63 Gy (whichever is the lowest) for the 24 patients of Figure 2. (b) TCP values for the constant 55 Gy prescription dose (blue) and the individualized *D*_presc_ shown in Figure 3(a). The increase in the mean TCP over the patient sample is obtained for no change in mean NTCP (adapted from Malik et al. (2007) with the TCP values recalculated using the parameters in [37]).

The basic idea is illustrated in Figures 2 and 3. The standard Clatterbridge protocol for the radical radiotherapeutic treatment of non-small-cell lung tumours was, until around 2009, to treat to a total dose of 55 Gy in 20 fractions over 4 weeks [34]. A relatively straightforward 3-beam 6 MV technique was employed.  Figure 2 shows the NTCP values (grade 2 radiation pneumonitis) for a series of 24 patients treated around 2005-6; the NTCPs were computed from the DVHs of the paired lung minus the GTV using the Lyman-Kutcher-Burman (LKB) NTCP model [23, 24] with parameters from [35]; naturally correction was made for the varying fraction sizes in the DVHs, using *α*/*β* = 3 [18]. The wide spread in these values is immediately evident, from as low as *≈*2% to as high as *≈*22%, corresponding to the large differences in normal lung coverage by the beams, caused by patient-to-patient differences in tumour size and location. In no sense could it be said that all these patients were “treated to tolerance.” Local control rates were estimated to be in the region of 35% [36].

For the same cohort, the prescription doses were recomputed so that the NTCP for each case was 10%. For a small number of the treatment plans, *D*_presc_ was reduced below its “isotoxic” value due to the oesophageal constraint (set at *D*_max⁡_ = 63 Gy for 20 fractions). An additional constraint we imposed was not to allow the TCP to exceed 99.0%; this restricted the highest value of *D*_presc_ to around 85 Gy. In all cases the number of fractions remained at 20. The “spectrum” of prescription doses is shown in Figure 3(a). In only two cases did this remain at *≈*55 Gy.  Figure 3(b) shows the TCP values “before” (pale blue) and “after” (maroon) dose customization, estimated using the “Marsden” TCP model [21, 22] with parameters from [37] (see below). The spread in TCP at the constant *D*_presc_ of 55 Gy is due to the variation in GTVs, which is translated into initial clonogen number *N*_0_ via the clonogen density *ρ*_clon_. The TCPs for the “isotoxic” prescription doses range from *≈*5% to *≈*100% (to be precise, 99%). The really important number is the average TCP which has increased from *≈*44% to *≈*60%. This has been achieved for absolutely no increase in the average NTCP, which is *≈*10% in both cases. In clinical practice the resulting increase in the average local control rate may be even greater, as the *reduction* in *D*_presc_ for a number of patients in this modelling exercise would probably not be applied clinically.

The parameters used in the TCP estimation were *N*_0_ = 10^7^ clons cm^−3^; $\overset{-}{\alpha }=0.307$ Gy^−1^, *σ*_*α*_ = 0.037 Gy^−1^, *α*/*β* = 10 Gy; *T*_dbl_ = 3.7 days; *T*_delay_ = 20.9 days. These were derived by fitting the predictions of the TCP model to published clinical outcomes [37] for a wide range of fraction sizes, total doses, and overall treatment times [36, 39, 40]; the fit was excellent.

### 3.2. Treatment Protocols Based on Level-I Optimization

In the UK two current phase I/II trials for radical radiotherapy of NSC lung tumours have been developed based on “isotoxic” dose individualization. The IDEAL-CRT trial [34, 43] involves 30 daily fractions over 6 weeks, with the prescription dose *D*_presc_ adjusted until NTD_mean_ (mean normalized total dose) for the non-involved lung is equal to 18.2 Gy; this corresponds to a 20% rate of grade 2, or greater, pneumonitis. *D*_presc_ is then reduced by 10% to compensate for the possible effect of the concurrent chemotherapy. additionally, *D*_presc_ is restricted to a window of 63–73 Gy. 

The I-START (ISoToxic Accelerated RadioTherapy) protocol [44] is a 20-fraction, 4-week treatment, radiation alone, and stages II to IIIb NSCLC patients are eligible. *D*_presc_ is chosen such that NTD_mean_ = 17.0 Gy and is restricted to the range 58–65 Gy, unless further limited due to cardiac, spinal cord or oesophageal constraints. This can be compared to the standard Clattebridge protocol of 55 Gy in 20 fractions for this patient group. It should be noted that both protocols are for fixed numbers of fractions; consequently increasing *D*_presc_ means that the dose per fraction is also increased.

Van Baardwijk et al. [33] described an individualized dose prescription study of 166 Stage-III NSCLC patients. Patients were treated to the maximally tolerable dose (MTD) by *increasing the number of fractions* (of 1.8 Gy twice daily) until normal-tissue constraints for the noninvolved lung and spinal cord were met. They reported favourable 1- and 2-year overall survival with acceptable toxicity. 

One of the clear advantages of this type of optimization is that improvements in the degree of conformality of treatment plans for any given tumour type, due, for example, to moving from 3-D conformal to intensity modulation, from fixed, few-field IMRT to rotational IMRT (Tomotherapy, RapidArc, VMAT, etc.), or even from megavoltage photons to protons, are automatically translated into increases in the target dose, and therefore into probable improvements in clinical outcome.

### 3.3. Level-II Optimization: Customization of the Prescription Dose and Fraction Number under “Isotoxicity”

**Figure 4:**
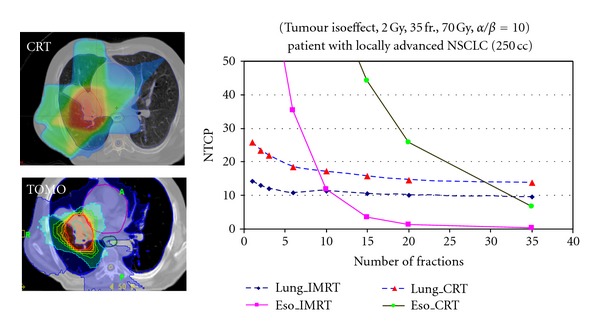
The variation of NTCP for radiation pneumonitis and oesophagitis, respectively, as a function of the number of fractions for a NSC lung radiotherapy case planned using tomotherapy (IMRT) and 3-D conformal techniques. The total dose is adjusted for tumour isoeffect using *α*/*β* = 10. The increase in oesophageal NTCP is consistent with the conventional application of the Withers formula [41], but the near constancy of lung NTCP is definitely not. Note also that the NTCP values are consistently lower for the more conformal tomotherapy plan (from [42]).

Fractionation is possibly the most “radiobiological” of all the variables in external-beam radiotherapy [45, 46]. The classic textbook recipe is that as small a fraction size as is practicable should be used to maximize the “therapeutic ratio” (see Figure 1), which follows logically if *α*/*β* is low for late-reacting normal tissues and high for tumour clonogens [46–48]. However, two issues complicate this oversimplified picture. Firstly, increasing the number of fractions may well take the overall treatment time beyond 3 weeks, and therefore, in the case of lung and head and neck tumours, into the clonogen proliferation time zone [40], leading to a loss of local control for a given tumour BED [49]. Secondly, the LQ-based “Withers” formula for computing *normal-tissue* isoeffect as a function of *α*/*β*, dose per fraction and total dose [41, 50] is frequently used in an illogical manner. If instead of setting the dose in the Withers' expression equal to the (tumour) prescription dose—that is, the “textbook” procedure—it is chosen to be representative of the “behaviour” of the normal tissue to which it is intended to apply (e.g., close to the mean organ dose for the case of “parallel” lung), then it can be shown that much larger fraction sizes can be safely used, especially for highly conformal treatment plans [42, 51].  Figure 4 illustrates this for the case of a lung tumour surrounded by “parallel” lung tissue; similar curves can be found in Vogelius et al. [52].

**Figure 5:**
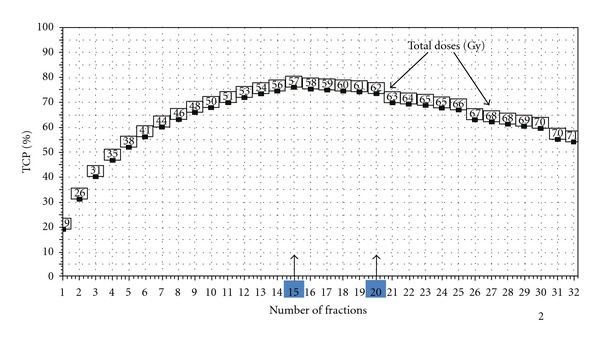
The variation of TCP with number of fractions, where the total dose (the numbers in the little squares) is adjusted to keep maintain isotoxicity, in this case 10% NTCP for the endpoint of grade 2 radiation pneumonitis (for parameters see Figure 2). The plot was generated by the BioSuite software [53]. In this case the standard prescription of 55 Gy in 20 fractions yielded TCP = 48%, NTCP = 6.6% (BioSuite is available from julien.uzan@clatterbridgecc.nhs.uk).

**Figure 6:**
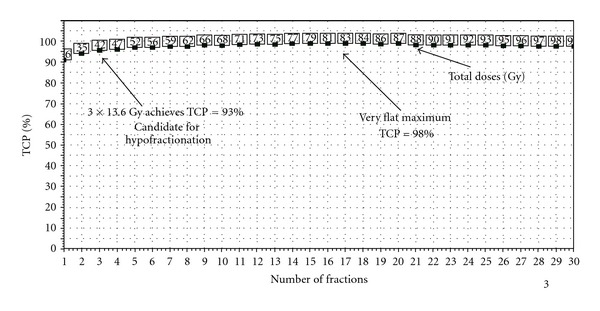
The variation of TCP with number of fractions, where the total dose (the numbers in the little squares) is adjusted to keep maintaining isotoxicity, in this case 10% NTCP for the endpoint of grade 2 radiation pneumonitis (for LKB parameters see Figure 2). The plot was generated by the BioSuite software [53]. In this case the standard prescription of 55 Gy in 20 fractions yielded TCP = 50.4%, NTCP = 4.3% (BioSuite is available from julien.uzan@clatterbridgecc.nhs.uk).

The BioSuite software [53] enables the impact of changing the number of fractions over a wide range to be explored. Figures 5 and 6 have been generated using BioSuite's “isotoxic optimization” option, with the choice of 10% NTCP for radiation pneumonitis (RP), for two contrasting treatment plans for NSC lung tumour radiotherapy. For the case of Figure 5, for the (standard) prescription of 55 Gy in 20 fractions, the TCP was 48.0% and the NTCP (RP) 6.6%. When the (total) dose is increased to make NTCP(RP) = 10%, we see how the TCP goes through a maximum at around 15 fractions and then decreases, due to clonogen proliferation (*T*_delay_ = 21 days, *T*_dbl_ = 3 days assumed). The optimum number of fractions is thus 15, that is, a 3-week treatment. This figure represents the “classical radiobiology” behaviour of the therapeutic ratio for a proliferating tumour (e.g., [49, 54]).

The treatment plan analysed in Figure 6 is also for a NSC lung tumour, but here the tumour is much smaller and more favourably located. The coverage of the non-involved lung is much less and this results in a very different dependence of TCP on the fraction number. This is a clear case of the effective dose in the normal lung being very much lower than the tumour dose, thus completely invalidating the conventional use of Withers' isoeffect formula (see the discussion at the beginning of this section).

The examples in the above two figures illustrate very clearly how different one patient can be from another in terms of the dependence of TCP on the number of fractions under lung isotoxicity. This strongly suggests that when the principal organ at risk behaves in a “parallel” fashion (as does the lung [55]), and there is evidence for tumour clonogen proliferation [40], biologically optimized radiotherapy will result in customizing the fractionation scheme based on the patient's treatment plan. The extreme hypofractionation employed in so-called stereoablative radiotherapy (SABR, formerly known as SBRT) for early stage NSC lung tumours [56] is effectively an example of this, though the dose is not generally individualized. However, for many of the reported series of SABR treatments local control rates are so high for acceptably low complication rates that it could be argued that there is no need for isotoxic dose customization [57, 58].

Hoffmann et al. [17] conducted an *in silico *trial, not dissimilar to the one described in the previous section (Figures 2 and 3). They individualized the dose prescription taking into account dose constraints not only for lung but also for spinal cord, oesophagus, brachial plexus, and heart. The number of fractions was set at 15, 20, and 33. They found that dose escalation was possible in 79% of the cases. They emphasised that to take the tumour dose to even higher levels it was essential to employ techniques which maximally spare the oesophagus.

### 3.4. Level-III BIOP: Radiobiologically Guided Inverse Planning

No modification of the treatment plan, that is, of the relative dose distributions, is involved in Level I or Level II optimization. Level III moves beyond this and, in the context of intensity modulation, exploits the mathematical properties of the TCP and NTCP functions in creating the “inverse” plan. This approach has one obvious advantage over dose-volume-based approaches to inverse planning: the number of degrees of freedom available to the optimizer is significantly increased. The intensity of each beamlet comprising the plan becomes a variable which can be adjusted to produce the best possible plan, from a radiobiological point of view. Thus the optimizer is now free to find solutions such as a reduction in the target dose adjacent to a critical normal tissue coupled with a boost to other parts of the target volume [59]. Obviously the target volume dose will no longer be uniform, but the TCP function will automatically take care of the effect of “hot” and “cold” spots.

There are however a couple of hurdles to overcome before this method can be applied. Firstly, all TPS inverse optimization engines work by minimizing a global cost function which is a measure of how well a plan meets clinical requirements (e.g., [8, 60]). For dose-based planning, these requirements are formulated in terms of dose/volume limits (e.g., maximum dose of 50 Gy in OAR1, minimum dose of 64 Gy in target volume 2, etc.). In the case of radiobiological planning, however, the objective function to be minimized should contain TCP- and NTCP-based criteria, possibly in addition to dose/volume-based ones. One possibility is to define the objective function so that the optimizer will try to maximize TCP while keeping NTCP equal to or below some user-defined threshold. Another possibility is to let the optimizer find the dose distribution yielding the highest “uncomplicated tumour local control probability” also called *P*+ [61, 62]. The mathematical form of the objective function has a critical influence on the resulting dose distribution.

Secondly, inverse planning engines rely on some form of *gradient descent* algorithm to minimize the objective function (e.g., [60]). These algorithms can be trapped in local minima [8], corresponding to suboptimal plans, when the objective function possesses specific mathematical properties such as nonconvexity. While the functions for TCP and NTCP in the most widely used models are intrinsically nonconvex, mathematical transformations can help to make these functions amenable to gradient descent minimization (with limitations, see reference [63]). In practice, very few commercial TPS include this kind of optimization. When they do, radiobiological models are simplified to improve the reliability of the inverse optimization process [64] at the expense of the radiobiological therapeutic ratio.

**Figure 7:**
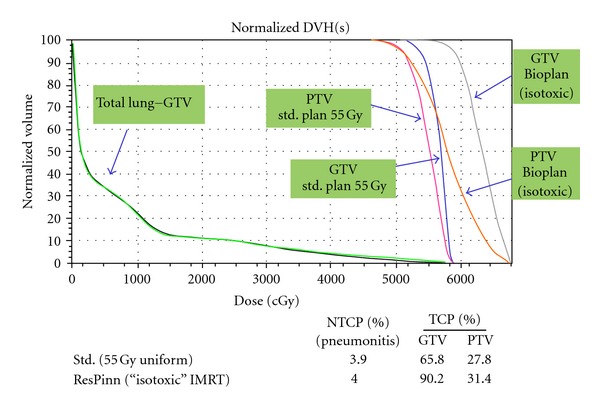
Dose-volume histograms for a radiobiologically guided inverse plan (3-field IMRT) aiming to maximize TCP for the same NTCP (labelled as “Bio-Plan (isotoxic)”) compare to a standard (3-field) treatment plan delivering 55 Gy (in 20 daily fractions) to a NSC lung tumour. The target volume (PTV, GTV) doses are more heterogeneous in the radiobiological plan. The increases in TCP values for the standard and radiobiologically optimized plans are also shown; the TCP parameters are from Nahum et al. [37]—see the subsection on “Level I” BIOP.

We give below an example of radiobiological optimization, for the case of a non-small cell lung tumour. This was created by ourselves by coding an objective function involving the Marsden TCP and LKB NTCP models inside the Pinnacle Research Interface (PHILIPS Oncology Systems) [65]. The parameters used were the same as in the “Level-II” example described earlier. The resulting (cumulative) DVHs are shown in Figure 7.

In searching for the highest TCP for a fixed NTCP the optimiser creates beams with a higher intensity in the centre (not shown explicitly here) resulting in nonuniform distributions in the PTV and the GTV; the corresponding DVHs are labelled as “Bio-Plan (isotoxic)” in the figure. There is a significant TCP increase while the DVHs for the “paired” lung minus the GTV (“uninvolved” lung) for the standard and radiobiologically optimized plans are virtually identical, thus demonstrating that the optimizer was able to keep the NTCP virtually constant. The much lower TCP calculated for the PTV, which is a somewhat artificial result, is a result of two factors: firstly and obviously the PTV volume is considerably larger than that for the GTV thus automatically reducing the TCP, but secondly the dose inhomogeneity in the PTV is very much greater. This serves to emphasize that if this strategy is to be implemented clinically then it will be very important to minimize tumour motion, due principally to respiration. Witte et al. [66] describe a sophisticated implementation of level-III BIOP involving not only TCP and NTCP but also the effect of random and systematic errors; their software platform was also the Pinnacle Research Interface. 

Engelsman et al. [67] showed that departing from a uniform dose to lung tumours could result in an increase in the therapeutic ratio. Their approach was much less sophisticated than radiobiological inverse planning; they explored the effect of reducing the field sizes, and thus PTV-CTV margin, thereby causing the dose in the tumour to be inhomogeneous. Simultaneously they increased the beam monitor units for all the beams in order to keep the NTCP constant. The tumour EUD, which is closely correlated with the TCP, went through a maximum at a margin size far below the one generally recommended. Similar results were obtained by Baker et al. [68] and by Popescu et al. [69]. In the latter case 4DCT images, reconstructed over the respiration cycle, were used as input to the Pinnacle research TPS, which accumulated the doses in each voxel as the images were deformed by respiration. It was found that the margin width yielding the *highest* TCP for *constant* NTCP for the uninvolved lung (all the doses were rescaled for each case) was around 5 mm, compared to the standard clinical choice of 15 mm. The above three approaches could be classed as “forward-planned radiobiological optimization,” though they belong to “Level III” as they involve modifications to the treatment plan in order to increase the TCP. 

A fixed number of fractions has been assumed in these examples of level-III radiobiological optimization. This need not be the case, though it is difficult to see how the number of fractions, *n*_frac_, can be added as an independent variable in the inverse planning procedure, as the values of TCP and NTCP for a given dose depend on *n*_frac_. This means that the inverse optimization would need to be performed several times, varying *n*_frac_ each time. One possible strategy to reduce the number of iterations could be to choose a standard number of fractions, say 20, perform the radiobiological optimization, and export the DVHs to *BioSuite*. Then a TCP versus *n*_frac_ curve (similar to those of Figures 5 and 6) could suggest an optimum value of *n*_frac_ which could be fed back to the TPS and the inverse radiobiological optimizer then re-run with this modified numbers of fractions. 

### 3.5. Level IV BIOP—Adding Patient-Specific Information from Functional Imaging

Currently a great deal of attention is being devoted to how information on individual tumour “biology” revealed by function imaging can be used for modifying the dose to the specific regions of the target (e.g., [69–73]). If the particular tumour property being imaged is related to clonogen density then it makes obvious sense to boost the dose to the identified subvolumes. Similarly if the images can be translated into the degree of hypoxia then the radiosensitivity in the TCP model can be suitably modified. The descriptive term “dose painting” has entered the radiotherapy lexicon to describe the various approaches being tried. 

**Figure 8:**
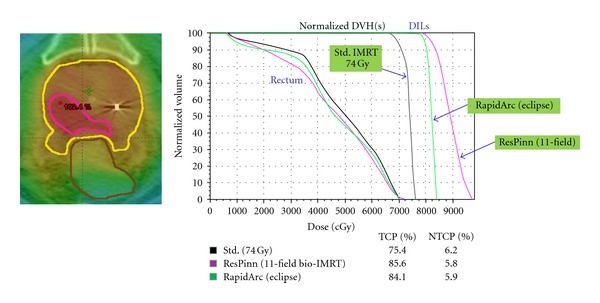
An example of a treatment under the Clatterbridge BIOPROP radiobiologically optimized prostate dose-painting protocol. The DIL is shown in pink on the left. The DVHs for the standard and dose painting plans are shown on the right. The TCP values are computed assuming that all the clonogens are contained in the DIL (or that any clonogens outside are 100% controlled). The NTCP values shown in the table correspond to rectal bleeding.

We describe below our own work with intermediate- and high-risk prostate tumours. The dominant interprostatic lesions (DILs) are identified using a combination of diffusion-weighted MR, choline PET and template biopsies. Our BIOPROP protocol makes use of our PRI-based radiobiological inverse planning (see previous section on Level III BIOP). The objective function attempts to maximise the TCP in the DILs (see Figure 8) while keeping the NTCP for the rectum below 7%, for each of two distinct endpoints, bleeding and faecal incontinence. Additionally there are physical dose constraints of 74 Gy minimum dose outside the DILs but inside the PTV and a maximum urethral dose of 74 Gy. To date we have delivered a small number of pilot treatments in 37 fractions.

The Pinnacle Research Interface (PRI) cannot be used clinically. Prostate tumours are currently treated at Clatterbridge using *RapidArc* (VARIAN) which is a form of rotational IMRT [9] planned on the Eclipse (VARIAN) TPS [74]. Consequently we wanted to deliver our inverse radiobiologically optimised dose painting plans using* RapidArc*. Our solution was to transfer the PRI-derived DVHs to the Eclipse TPS and use these to drive the *RapidArc* optimizer. An example of the resulting DVHs is shown in Figure 8.

It can be noted that the DIL DVH for the RapidArc plan corresponds to a more homogeneous dose (between 80 and 83 Gy) than that for the Pinnacle plan, which was created using 11 fixed IM fields. This is because it was not possible to ask the RapidArc optimizer to “maximize the TCP”. However, the effect is only to reduce the TCP by 1.5%. Our analysis of the first 5 BioProp treatments indicates an average TCP = 84.7% corresponding to maximum doses in the DILs from 82.4 to 87.4 Gy, compared to TCP = 70-71% for the standard 74 Gy treatment (parameters assumed in the TCP model: $\overset{-}{\alpha }=0.262$ Gy^−1^, *σ*_*α*_ = 0.045 Gy^−1^, *α*/*β* = 10, *ρ*_clon_ = 10^7^ cm^−3^; these were obtained from a fit to the clinical outcomes reported by Dearnaley et al. [75]).

### 3.6. Level V BIOP—Adding Individual Patient Biology to any of the above Levels

As far as we are aware there are no specific examples in the literature of either *in silico *or clinical studies of this further refinement of radiobiological optimization strategies. As discussed earlier, the currently used TCP and NTCP models are hybrids in the sense that whilst they make predictions for a specific treatment plan (to be precise for the DVHs computed from the plan) they effectively do so for a population of patients with that specific treatment plan. Thus what TCP = 65% means is that 65 out of 100 patients will have a tumour of that size controlled by the dose distribution in question. If, however, we knew the radiosensitivity of the tumour clonogens for the patient undergoing treatment [20], then this prediction would be overwhelmingly converted into either one or zero that is, controlled/not controlled, with the TCP increasingly rapidly from zero to unity over a very narrow range of doses [18, 21, 22]. This information would then enable us to choose the lowest prescription dose still achieving say TCP > 99%. Equally well, a prediction of a complication rate of say 6.0% means that for that specific dose distribution in the normal tissue in question, 6 out of 100 patients will experience the complication. Were we to possess information on the individual biology of the patient this prediction would in principle be narrowed down to either a very low (possibly zero) probability or a very high one. These issues have been explored by, amongst others, Lambin et al. [76] and Rutkowska [77].

Tucker et al. [78] showed that single nucleotide polymorphisms (SNPs) can significantly improve the ability of the Lyman MLD model to predict the incidence of radiation pneumonitis. In a study on clinical risk factors the same group showed that the generalized Lyman model with patient smoking status taken into account produced NTCP estimates up to 27 percentage points different from the model based on dose-volume factors alone [79]. Valdagni et al. [80] attempted to understand why, despite “excellent” rectal dose-volume histograms (DVHs), certain patients treated for prostate cancer exhibited late rectal bleeding (LRB) whereas others with “poor” DVHs did not. Thirty-five genes involved in DNA repair/radiation response were analyzed. It was found that nine genes were significantly down-regulated in the low-risk bleeder group versus the high-risk bleeder and high-risk non-bleeder groups; four genes were significantly upregulated in the high-risk non-bleeder group compared to the other groups. It is to be hoped that studies such as these will result in the NTCP models capable of making different predictions for “biologically different” patients having very similar DVHs, and ultimately in the incorporation of such improved models into the various levels of radiobiological optimization discussed here [81, 82].

## 4. Summary and Conclusions

The recent AAPM task group report on the use and QA of biologically related models for treatment planning [83] stated in the Introduction:

*Until recently, the quality of a RT plan has been judged by physical quantities, that is, dose and dose-volume (DV) parameters, thought to correlate with biological response rather than by estimates of the biological outcome itself. It is widely recognized that the DV criteria, which are merely surrogate measures of biological responses, should be replaced by biological indices in order for the treatment process to more closely reflect clinical goals of RT. Developments in our understanding of advantages and limitations of existing dose-response models begin to allow the incorporation of biological concepts into a routine treatment planning process.*

This paper has proposed several ways (or levels) in which the “biological indices” TCP and NTCP can be incorporated directly into the treatment planning process, not merely in order to evaluate and compare rival plans (e.g., Iori et al. [84]), but to *optimize* treatment plans in terms of the prescription dose and number of fractions, thereby improving clinical outcomes. As a result of much careful research, summarized in the QUANTEC (QUantitative Analysis of Normal Tissue Effects in the Clinic) series of papers [85], the predictions of especially the widely applied and researched Lyman-Kutcher-Burman NTCP model [23, 24] are now sufficiently reliable for certain important organs/tissues and endpoints (rectal bleeding [86], pneumonitis [55], radiation-induced liver disease, xerostomia in the parotid glands, possibly also cardiac complications [87]) that treatment protocols involving “isotoxic” tumour dose individualization can be developed and applied [17, 33, 34, 44]. Software such as *BioSuite *[53] makes this straightforward as long as only prescription dose *rescaling* and/or fraction number customization is involved. In order to plan and deliver radiobiologically based IMRT, however, treatment planning systems must incorporate TCP and NTCP into the objective functions; at the time of writing full-blown radiobiological inverse planning is possible in at most two of the commercial systems [64, 83], though planning based on gEUD [27] is more widely available.

By taking full advantage of the steadily improving degree of conformality achievable with modern techniques, the individualization of fractionation (towards fewer fractions) has huge radiobiological potential. Furthermore, hypofractionation also delivers increased patient convenience and a reduction in cost per treatment course, which could be especially important for proton therapy [13]. Two notes of caution are in order however. Firstly the methods and models employed to generate much of the data in this paper (e.g., Figures 4, 5 and 6) are either directly, in the case of the Marsden TCP model [18, 21, 22, 31], or indirectly, in the case of Lyman-Kutcher-Burman NTCP model [18, 23, 24], based on the linear-quadratic expression linking (cell) surviving fraction and absorbed dose [41, 45–50, 88]. At doses per fraction above *≈*10 Gy, however, the so-called *generalized* linear quadratic model proposed by Wang et al. [89] and by Carlone et al. [90] may be more correct, though this is by no means universally accepted [91]. Whatever the “truth” eventually turns out to be, if the LQ model does overpredict cell killing at very large fraction sizes then at such doses per fraction LQ-based radiobiological models will result in an *over*prediction of NTCP but an *under*prediction of TCP. Consequently the resulting hypofractionation schemes are highly unlikely to cause excessive complication rates [58]. Secondly, regarding proton-beam radiotherapy there is an added complication that is (thankfully!) not present when considering low-LET modalities (which include the bremsstrahlung photon beams produced in linear accelerators): the biological effect of a given number of Grays of absorbed dose is not the same everywhere in the patient (or in a phantom); it depends on the proton energy spectrum [92–94]. Though this dependence of proton RBE on energy and hence on depth, and therefore position in the patient, probably never exceeds *≈*10%, Dale et al. [93] have pointed out that a 10% uncertainty in RBE is precisely equivalent to a 10% uncertainty in dose in photon treatment plans, which would not be tolerated. Therefore more research is required on proton RBE variation [92, 93]. 

In conclusion, if today's sophisticated imaging, treatment planning and radiation-delivery techniques, and tomorrow's genome-based patient biology are to be translated into maximum clinical benefit then the stipulation of a fixed dose to the target volume in today's treatment protocols must be replaced by *individualized doses*, and, in certain situations, *individualized fractionation*. In the right hands biomathematical models of radiation effect are powerful tools [95, 96]; to paraphrase Chapman and Gillespie [97], let's use them!
